# Sperm selection in natural conception: what can we learn from Mother Nature to improve assisted reproduction outcomes?

**DOI:** 10.1093/humupd/dmv042

**Published:** 2015-09-19

**Authors:** Denny Sakkas, Mythili Ramalingam, Nicolas Garrido, Christopher L.R. Barratt

**Affiliations:** 1 Boston IVF, 130 Second Ave, Waltham, MA 02451, USA; 2 Reproductive and Developmental Biology, Medical School, Ninewells Hospital, University of Dundee, Dundee DD19SY, UK; 3 IVI Valencia, Guadassuar 1 Bajo, 46015 Valencia, Spain

**Keywords:** Fallopian tube, spermatozoa, uterine tube, uterus, vagina, sperm selection

## Abstract

**BACKGROUND:**

In natural conception only a few sperm cells reach the ampulla or the site of fertilization. This population is a selected group of cells since only motile cells can pass through cervical mucus and gain initial entry into the female reproductive tract. In animals, some studies indicate that the sperm selected by the reproductive tract and recovered from the uterus and the oviducts have higher fertilization rates but this is not a universal finding. Some species show less discrimination in sperm selection and abnormal sperm do arrive at the oviduct. In contrast, assisted reproductive technologies (ART) utilize a more random sperm population. In this review we contrast the journey of the spermatozoon *in vivo* and *in vitro* and discuss this in the context of developing new sperm preparation and selection techniques for ART.

**METHODS:**

A review of the literature examining characteristics of the spermatozoa selected *in vivo* is compared with recent developments in *in vitro* selection and preparation methods. Contrasts and similarities are presented.

**RESULTS AND CONCLUSIONS:**

New technologies are being developed to aid in the diagnosis, preparation and selection of spermatozoa in ART. To date progress has been frustrating and these methods have provided variable benefits in improving outcomes after ART. It is more likely that examining the mechanisms enforced by nature will provide valuable information in regard to sperm selection and preparation techniques *in vitro*. Identifying the properties of those spermatozoa which do reach the oviduct will also be important for the development of more effective tests of semen quality. In this review we examine the value of sperm selection to see how much guidance for ART can be gleaned from the natural selection processes *in vivo*.

## Introduction

In 1992 a landmark study that recovered artificially inseminated spermatozoa from the Fallopian tubes of a woman undergoing total abdominal hysterectomy showed that of the hundreds of million sperm deposited in the female tract only a thousand or less are recovered from the Fallopian tubes (Williams *et al.*, 1992). This dramatic reduction in numbers clearly highlights the variety of hurdles that sperm must overcome in order to reach their final destination before one fertilizes the egg.

These obstacles include the acidic nature of the vagina, cervical mucus, the entrance to the cervix, the narrowness of the uterotubal junction, the response of the immune system, etc. The concept is of a physiological screening process that allows only a selected few sperm to reach the site of fertilization. Stringent selection mechanisms will reject all but a significant minority of the spermatozoa released at ejaculation. Perhaps not surprisingly evidence in human for these stringent mechanisms is minimal and some valid questions remain unanswered. For example; Are the cells that have successfully traversed the cervix the most motile cells in the ejaculate? Are cells in the oviduct more fecund than cells in the uterus? We do not really know the answers to these questions. The presumption is that the oviduct population of cells is highly fecund, but there is a marked paucity of data. In fact, with the advent of assisted reproductive technologies (ART) and in particular intra cytoplasmic sperm injection (ICSI), the identification of characteristics encompassed by the ‘best’ male gamete are not always deemed important to search for and when preparing sperm the samples are fundamentally mistreated compared with the care we take with eggs.

In natural conception only a few sperm cells reach the ampulla or the site of fertilization. However, whether this population is a more fecund group of cells compared with other motile cells that are ejaculated, or those that successfully transverse the cervix is unknown (Williams *et al.*, 1993). In animals, some studies show that the sperm recovered from the uterus and the oviducts have higher fertilization rates (Cohen and McNaughton, 1974; Fischer and Adams, 1981; Siddiquey and Cohen, 1982) compared with ejaculated cells but this is not a universal finding. In this review we examine some of the *in vivo* selection processes to see whether they could be exploited for the improvement of laboratory tests of sperm quality and how they relate to current and future sperm selection strategies.

## Methods

A review of the literature examining characteristics of the spermatozoa selected *in vivo* is compared with recent developments in *in vitro* selection and preparation methods. PubMed was used to search the MEDLINE database for peer-reviewed original articles and reviews. Searches were performed but not limited to using key words such as sperm selection, sperm transport, sperm preparation in conjunction with ART, IVF, IUI, nature, natural, Fallopian tube, uterine tube, uterus, vagina and oocyte. Where possible historical references were also collected from articles when referring to original experiments in books or book chapters. The most relevant publications were discussed, assessed and selected. Contrasts and similarities are presented.

## Natural sperm selection: the voyage in the tract and to the egg

### Dynamics of sperm transport

In animals, passage of sperm through the female reproductive tract is regulated to maximize the chance of fertilization (Hunter, 1981). In humans there are some data supporting this as oocytes are usually fertilized within hours of ovulation (Harper *et al.*, 1994). In some species however, sperm may be inseminated days or even months before the arrival of the oocyte. In humans, fertilization occurs when intercourse takes place up to 6 days before ovulation (Wilcox *et al.*, 1995), therefore spermatozoa are capable of surviving a relatively long time in the female tract. Sperm must somehow use their limited resources and/or exploit those of the female tract to maintain their fertility in the face of numerous impediments (reviewed by Suarez and Pacey, 2006; Mortimer *et al.*, 2013).

### Sperm entry and distribution in the vagina

The site of semen deposition is species-specific. In humans, semen is ejaculated near the anterior vagina near the cervical opening. Spermatozoa encounter a hostile vaginal pH and immune responses at the site of deposition (Boskey *et al.*, 2001). After semen deposition in the anterior vagina there is substantial sperm loss (Baker and Bellis, 1993). In a 5 year study of 11 female volunteers Baker and Bellis (1993) examined the characteristics of sperm loss from the vagina following coitus. They found that flow back occurred in 94% of copulations with the median time to the emergence of flow back of 30 min. Within minutes of vaginal deposition, human sperm begin to leave the seminal pool and swim into the cervical canal. Sperm were found in the endocervix from 90 s to 3 min after ejaculation (Sobrero and Macleod, 1962). During sexual intercourse, the initial sperm-rich fraction of the ejaculate may come into contact with cervical mucus extending into the vagina with the rest of the fluid remaining as a pool in the vagina. During ejaculation the first fractions voided are mainly sperm-rich prostatic fluids (Bjorndahl and Kvist, 2003). In humans, the sperm-rich fraction coalesces into gelatinous lumps which are hygroscopic and may act to prevent the sperm-rich fraction from being expelled after coitus. The coagulate is formed within about a minute of coitus, acts in a similar way to a cervical plug (Harper, 1994) and is then enzymatically degraded (Lilja and Lundwall, 1992). It has been proposed (though not shown) that this coagulum serves to hold the sperm at the cervical os and that it protects sperm against the harsh environment of the vagina. Human sperm must contend, however briefly, with the acidic pH of vaginal fluid and the female immune system. However, within a short period of time thousands of sperm reach the Fallopian tubes (Overstreet and Cooper, 1978) and thereby escape the significant cellular immune response in the female reproductive tract (Sharkey *et al.*, 2012). In contrast, in the laboratory setting, the entire ejaculate is collected in one container and the interaction of spermatozoa with seminal plasma is very different, as spermatozoa are trapped in the coagulum during liquefaction. This coagulum is subsequently liquefied by the action of prostatic proteases, during which time its osmolality rises (Bjorndahl and Kvist, 2003). In addition, once the ejaculate is mixed into a media it may in turn also detract from one of the functions of seminal plasma, which is to kill poor quality sperm.

In some animal species, such as the pig, sperm are deposited directly into the uterine cavity. This bypasses the vaginal barrier and makes the sperm available to the Fallopian tubes more easily (Hunter, 1981). In some rodents the sperm deposited in the vagina forms a plug and this can extend for the whole length of the cervix which in turn can prevent retrograde loss of the sperm from the site of deposition (Blandau, 1945).

### Sperm entry and distribution in the cervix

Human sperm enter the cervical canal rapidly where they encounter cervical mucus. Around ovulation the cervical mucus is optimal for sperm passage with highest antibacterial action and low vaginal pH. Sperm can spread remarkably quickly (Katz *et al.*, 1989) depending on the viscosity of the cervical mucus. In midcycle the cervical mucus becomes extremely hydrated (Katz *et al.*, 1997) and this allows greater penetrability to sperm (Morales *et al.*, 1993). The progressive motility of the spermatozoon is essential for it to pass through the cervical mucus and hence spermatozoa with poor motility and concomitantly with abnormal morphology are filtered out during this passage. This is thought to be one (gross) form of sperm selection. Penetrating cervical mucus is a substantial barrier to sperm migration and it depends on seminal enzymes, external forces due to visceral contractility (Katz *et al.*, 1989) and the hydration of the mucus which varies with the menstrual cycle (Wolf *et al.*, 1978), as well as sperm number and motility. Yudin and colleagues (1989) reported that the cervical mucus architecture is more compact at its borders making it more challenging for the sperm (Yudin *et al.*, 1989). In 1982, Mortimer and Templeton (1982) confirmed the existence of a selection for morphologically normal human spermatozoa within cervical mucus. Conversely, although they provided evidence for a higher proportion of normal cells in the oviduct compared with the ejaculate (Mortimer *et al.*, 1982) they also suggested that ‘abnormal’ spermatozoa may reach the site of fertilization which is in accordance with findings in other species. However, detailed evaluation of the morphology of spermatozoa recovered from the cervical canal shows that the selection of spermatozoa is largely achieved by reductions in spermatozoa with midpiece, tail, and other defects which might be expected to impair their motility (Mortimer, 1994).

Sperm have been observed trapped in human cervix epithelial crypts (Croxatto, 1995; Hunter, 1995), though the mechanics of trapping and release is unexplored. This trapping may produce a reservoir, slowly releasing sperm into the reproductive tract over several days, but this is a concept not a mechanism (Suarez, 2002). The time it takes for the journey through the cervix and whether sperm remain there is not precisely documented. Vigorously motile sperm have been recovered from the human cervix up to 5 days after insemination (Gould *et al.*, 1984). How and whether these sperm recovered from the cervix would continue their journey into the Fallopian tube and if they could reach the egg is not known. Cervical crypts are thought to entrap and store sperm (Fawcett and Raviola, 1994; Harper, 1994) and scanning electron microscopy of the human cervix indicates that mucosal grooves forming a preferential pathway for sperm could be present, though a comprehensive study of the human cervix is needed to determine whether sperm follow these grooves to traverse the cervical canal. Like the vagina, the cervix can mount immune responses to spermatozoa stimulating the migration of leukocytes, particularly neutrophils and macrophages, into the cervix as well as into the vagina (Barratt and Pockley, 1998). Evidence indicates that the leukocytic invasion serves to protect against microbes that accompany sperm and does not present a barrier to normal motile sperm, at least not shortly after coitus (Suarez and Pacey, 2006).

### Sperm entry and distribution in the uterus

On passing through the cervix, sperm enter the uterus. It is thought that sperm rapidly progress through this region, aided and directed by peristaltic contractions (Kunz *et al.*, 1996). Only a few centimetres in length, the human uterine cavity is relatively small and could be traversed in <10 min. Thompson and colleagues (1992) showed that sperm can be obtained by flushing the uterus 4 h post insemination, but only recovered low numbers of spermatozoa and were unable to determine what percentage of sperm passed from the cervix to the uterus. Transport of sperm through the uterus is aided by pro-ovarian contractions of the myometrium (Lyons *et al.*, 1991). In 1982 Templeton and Mortimer (1982) showed laparoscopic recovery of human sperm from the pouch of Douglas around the peri-ovulatory period in 45% of the patients studied. The study confirmed that there is usually a reduction in sperm numbers along the length of the female tract of between 5 and 6 orders of magnitude compared with the number of spermatozoa inseminated. It would be highly informative to investigate whether the current understanding of sperm migration and peristaltic flow are consistent with the transport of sperm to the uterotubal junctions. Sperm transport through, and interaction with the uterus, remains a poorly understood area.

### Sperm entry and distribution in the uterotubal junction

The lumen of the uterotubal junction is narrow and filled with mucus (Jansen, 1980). The physical interaction between human spermatozoa and the epithelium of the human Fallopian tube has only been investigated *in vitro* using a variety of techniques. The ‘live’ observation of human spermatozoa incubated with 1 day old cultures of tubal epithelium demonstrated that spermatozoa can show a strong physical interaction with epithelial cells (Pacey *et al.*, 1995b). These results are the first descriptions of sperm-epithelial ‘binding’ in the human. They are similar to other observations made in a variety of non-human mammalian species. It is suggested that this interaction may be an important feature of normal sperm transport in the human uterine tube *in vivo* (Pacey *et al.*, 1995b). Some of the animal studies show that normal morphology and motility are not sufficient for enabling sperm to pass through the junction. An additional factor, likely a sperm surface protein or proteins, is required by each sperm for it to pass through the junction (Nakanishi *et al.*, 2004). In animals including cows, pigs, rabbits and many other species (Hafez, 1968) it has been demonstrated that the junction of the uterus and tubes is more complicated compared with that of humans.

### The sperm storage reservoir in humans

A very distinct sperm reservoir has been identified in the oviducts of animals, for example hamsters, pigs, sheep (Yanagamachi and Chang, 1963; Hunter, 1981; Hunter and Wilmut, 1984), but not in humans. The *in vitro* studies to date show that a functional reservoir may exist, created by detaining human sperm in the tubal isthmus (Pacey *et al.*, 1995b). Such a reservoir could be created when sperm *in vitro* intermittently bind to the epithelium lining the tube. In humans motile sperm have been observed to bind via their heads to the apical surface of endosalpingeal epithelium *in vitro* (Pacey *et al.*, 1995a). It is interesting to note that sperm transport is slowed by the mucus in the Fallopian tube. The mucosal folds and architecture provide complexity for their transport in the tubes. In addition to this, sperm interaction with the tubal epithelium may slow down the advancement of possible abnormal spermatozoa. These innovative studies on improving our understanding of the interaction of sperm with the Fallopian tube have failed to translate into our current sperm preparation techniques. One problem may be that almost all data available on sperm interaction with the epithelium of the tract is from *in vitro* studies and as such the relevance to the journey of the spermatozoon *in vivo* remains a substantial question. New technologies such as microfluidics (Suh *et al.*, 2006) could however play some role in mimicking the journey of the sperm through the reproductive tract.

Overall, data of human sperm distribution in the Fallopian tubes of women have not provided a clear picture of the events of sperm transport (Williams *et al.*, 1993). Sperm recovered at various times in different regions of the Fallopian tube have varied so much in numbers that the data do not permit the construction of a model for the pattern of tubal sperm transport. Nevertheless, since pregnancy has been shown to result from intercourse as long as 6 days before ovulation (Wilcox *et al.*, 1995) human sperm must be stored somewhere in the female tract and the fact that human endosalpingeal epithelium prolongs survival of sperm *in vitro* indicates that the Fallopian tubes are strong candidates for functional storage sites.

## Natural sperm selection

### Evolutionary mechanisms used to promote sperm selection

There are a plethora of papers—almost exclusively based on animal studies—discussing sperm competition, defined as when more than one male has the opportunity to fertilize a single female during the same fertile period. A detailed analysis of sperm competition and the evolution of sperm function is covered in recent reviews (Fitzpatrick and Lupold, 2014; Lehtonen and Parker, 2014; Ramm, 2014). However, there are two key questions.

First, why are human sperm of such poor quality compared with most other animals? Humans have relatively low levels of sperm competition primarily because there is not intensive male–male competitive matings. Consequently there is less pressure to drive the rate and quality of sperm production. This is in contrast to species with high levels of multiple matings like chimpanzees where the testis size in relation to body mass is high, there are high sperm numbers and the cells are relatively homogenous (Ramm, 2014). Monogamous species have poorer sperm quality including a significant degree of heterogeneity of sperm function and structure (see (van der Horst and Maree, 2014). Humans thus probably produce poor quality spermatozoa due to the low risk of sperm competition but the quality of the cells is sufficient for effective reproduction.

Second, what can we learn from animal models as it relates to natural sperm selection in humans? The diversity of mating strategies (excluding of course external fertilization), differences in reproductive tracts, function etc. make comparisons very difficult. In reality more detailed human experiments are required. We have relied too heavily on the usual models such as mice which may have limited clinical relevance. Some interesting examples however exist, including the naked mole rat which is monogamous, has relatively poor sperm quality yet every estrus copulation provides multiple offspring (van der Horst *et al.*, 2011). This may illustrate what spermatozoon traits are good enough for fertilizing the oocyte.

Suffice it to say, in all mammalian species studied to date, of the many millions of sperm ejaculated a very small number appear to gain access to the site of fertilization. While there is a breath-taking paucity of information in humans the available data are consistent with only tens to hundreds of sperm at the site of fertilization (ampulla) at or near the time of ovulation (Williams *et al.*, 1993). The ampulla population of cells is clearly a highly selected group as, for example, only motile cells can pass through cervical mucus and gain initial entry into the female tract. However, whether this population is a special/more fecund population compared with other motile cells that are ejaculated, or successfully penetrate the cervix is unknown. If we use IVF as an example (albeit performed on subfertile rather than fertile couples) significantly higher fertilization rates were achieved with 20 000 sperm/egg versus 5000 sperm/egg (Tournaye *et al.*, 2002). This efficacy is significantly different to that occurring *in vivo* (hundreds of sperm/egg) and argues for a more fecund selected ampulla population compared to what we can, as yet, select/identify *in vitro*. Presumably if all cells were equally good then fewer sperm numbers per oocyte would be required. However, with so few *in vivo* studies in humans it is impossible to get robust conclusions. Additionally, the *in vivo* studies only represent one time point and as such many more cells may be present in the ampulla over the time period where the egg can be fertilized potentially bringing the number of cells/oocyte closer to the IVF range.

In humans there appear to be various lower threshold criteria (not absolute) for conception and higher numbers of cells inseminated (*in vivo*) led to increases in success (Publicover and Barratt, 2011). As such, part of the process of sperm transport is a numbers game (to achieve the necessary tens of sperm in the ampulla). However, there are such differences in the fecundity of men even when ‘reasonable numbers of motile cells’ are present that either the proportion of effective cells in the ejaculate/and or their fecundity is significantly different. Further examples of this relate to donor insemination where fecundity of donors can vary by a factor of 3 even though motile sperm numbers are equal (Barratt *et al.*, 1998).

## Assisted reproduction and sperm selection

As such we understand very little about the relative effectiveness of sperm populations and what makes them special or selected. In fact, despite decades of research, we are left with many more questions than answers. For example, in humans is there a ‘special forces’ (elite) number of cells that reach the oviduct that are selected/select themselves for transport for IVF? If this is correct, the ‘ampulla’ [oviduct] selected cells are special and thus examination of their characteristics is essential. Logically, if we were to use these cells (or those selected by the same mechanisms) in ART, the success rate (sperm/fertilized egg ratio) would approach *in vivo* levels. Alternatively, are all motile sperm capable of fertilization or is it a purely random event (presuming minimal capacity to fertilize in a single population)? Furthermore, why are the overwhelming majority of motile cells in subfertile men incapable of fertilizing an egg *in vitro* [at least as selected by normal IVF means]? Could this be corrected by selecting more fecund cells in the ejaculate?

## What are we missing and is sperm selection important?

ART have rapidly evolved since the first *in vitro* child was born in 1978. Nevertheless, we need to assume that treatments are still far from being 100% effective, as many patients fail to achieve pregnancy and others frequently need several attempts to achieve parenthood. Even when using the best gametes, obtained from young, healthy and previously fertile donors (Garrido *et al.*, 2011, 2012) it can take several embryos to be successful. In relation to the egg it has been reported that <7% of the retrieved oocytes lead to a live birth (Patrizio and Sakkas, 2009). From the male viewpoint, the improvements needed are closely linked with the need to establish robust sperm quality indicators, in order to use them as a diagnostic tool to increase the success of ART, and in order to design appropriate strategies for sperm treatment (thinking about sperm as a patient) or selection. Implementing such sperm diagnostic and/or selection techniques could significantly improve live birth rates, still acknowledging that outcomes will also depend heavily on oocyte and endometrium quality.

As discussed above the selection mechanisms that operate in nature are able to discriminate the quality of spermatozoa. Understanding these mechanisms will help clarify the properties of the selected spermatozoa and provide valuable insights required for the development of useful tests of semen quality, which, despite many years of development, remain elusive. A number of differences however need to be accounted for when comparing natural selection versus different ART. Ultimately the sperm quality used in ART declines, going from IUI, IVF to ICSI, so the selection criteria and applicability may change dramatically.

## The complexity of sperm: normal and abnormal

Sperm cells are by far one of the most specialized cells in the human body. They are designed to accomplish a very difficult mission, involving several consecutive phases, each one independent and highly critical. The different components of a spermatozoon each play a crucial role during the conception process (Gagnon, 1999), including the head containing DNA/chromatin that needs to be correctly condensed and decondensed at specific moments. The midpiece which contains the energy-generating mitochondria and the flagellum transforming energy into movement. In addition, sperm possess all the mechanisms of oocyte recognition, fusion, and intracellular structures and factors affecting early embryo development and division (Krawetz, 2005). All these procedures are dependent on critical molecules, whose evaluation, theoretically, could find a place in the future semen analysis given their physiological importance (Barratt *et al.*, 2011; Hwang *et al.*, 2011). Moreover, we must consider that the rules for a sperm to win, and their ‘technical specifications’ could be radically different in ART compared with the ones needed for natural reproduction (Garrido *et al.*, 2008). It is logical that if we can mimic the selection process that produces the sperm population able to arrive at the egg *in vivo* then we will have a much better understanding of male fertility.

Any sperm's capacity to succeed *in vivo* is dependent upon the weakest point in the chain of events. Therefore, it is very complex being a successful sperm, whereby for natural conception the cell will have to have the correct motility, structure and genomic apparatus. It therefore becomes difficult to precisely measure how successful a spermatozoon can be and, when ART are added to the equation, it is difficult to measure if all 3 of the above components (head, midpiece and tail) are required.

For example, there are elegant studies demonstrating that within the same ejaculate, there are genetically unique and very different cells that may ultimately influence embryo quality and the chance of fertility (Wang *et al.*, 2012). This is of paramount importance when considering sperm cell selection in assisted reproduction: success depends on one single cell, in comparison with other tissues or organs, where many cells work jointly and are genetically identical and a single cell cannot drive failure. Moreover, sperm cells exclude each other from developing their full tasks: the first entering the oocyte precludes the others' participation. If the winner is unable to conduct the next biological task required for success, then the entire sperm cohort is futile.

## Sperm quality assessment beyond the World Health Organization Guidelines

### What is missing with semen analysis?

Semen quality has traditionally been measured under a global viewpoint, following the recommendations established by the World Health Organization (WHO) (Cooper *et al.*, 2010; WHO, 2010). In the latest version, the minimal thresholds were readjusted to consider a sperm sample as normal or not, changing to some extent a sperm samples' categorization.

Semen analysis, when performed without adequate quality control, is of almost no clinical value. However, even when using appropriate methods, the predictive value of a semen analysis to forecast or categorize a male as fertile or not, is far from absolute with a considerable overlap between the values exhibited by males who conceive, and the subfertile males (Ollero *et al.*, 2001; Eliasson, 2010; De Jonge, 2012; Esteves *et al.*, 2012). Moreover, there is a potential wide variation among samples from the same individual. It is therefore clear that more sophisticated—functional testing—is urgently required.

### Inherent difficulties of relating sperm diagnosis to success in assisted reproduction

In the natural selection of sperm the process of arriving at the egg is a numbers game to achieve the necessary tens to hundreds of sperm in the ampulla. IVF drastically reduces the numbers game while ICSI removes it altogether. Trying to develop a diagnostic tool to determine a gamete's quality by relating a specific measurement with fertility success has a number of intrinsic limitations. As previously described (Garrido *et al.*, 2008), the aim of searching for diagnostic tools to determine sperm quality is complicated by the multifactorial nature of a successful pregnancy, and we are indeed aiming to forecast the combined importance of oocyte, sperm, endometrial environment and endometrium. There are even selection factors in the female reproductive system apart from the oocyte and uterus, for example structural uterine anomalies impeding sperm progression or factors compromising both sperm biology and interaction with ampullary mucous or even cilia. These could represent further variables that we currently define as idiopathic infertility. There are too many uncontrolled factors, which will undoubtedly introduce a significant bias in the quest to establish a strong correlation between sperm and a reproductive result.

## Current sperm selection techniques

**Figure 1:**
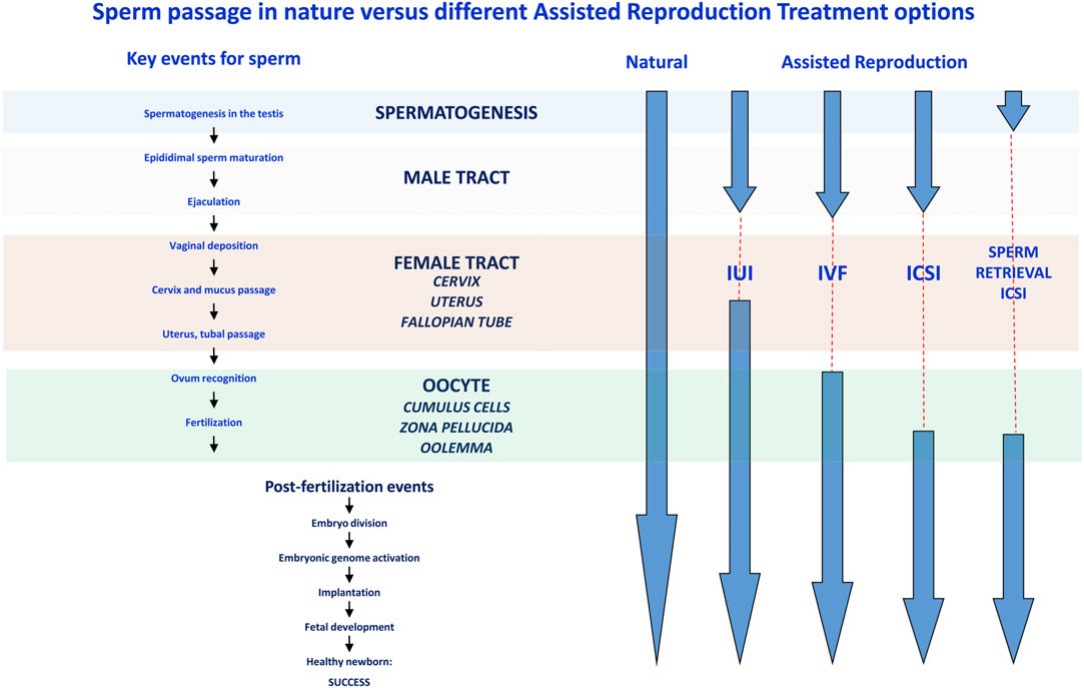
A comparison of sperm passage in nature versus different assisted reproductive technologies (ART). The key events encountered by sperm as they travel from the testes to the egg are highlighted on the left. The comparison between what the sperm encounters during natural conception and the different ART is shown on the right. The dashed lines represent the missing steps when comparing the different *in vitro* assisted reproduction process with the steps encountered by the sperm during natural conception. The more complex the ART procedure the greater the length of the dashed lines. IUI, intrauterine insemination.

The sperm selection techniques that are currently in routine use mostly rely on a brief motility challenge [swim-up] or a forced passage through a differential gradient. This challenge is a far cry from truly mimicking the natural selection characteristics seen *in vivo*. The new sperm diagnostic/selection technologies being developed are focussing more on the development of tests that both diagnose and select or deselect sperm on specific cellular characteristics. Broadly these techniques can be characterized as those that examine membrane integrity, density, surface charge or morphological characteristics. Some tests attempt to mimic a particular ‘natural’ process while others have been adapted for use in ART and do not even attempt to mimic a natural process (Fig. 1). The following sections discuss these relatively new technologies and investigate where they do or do not align to natural selection. They also briefly review their potential although many have not been stringently tested in large clinical trials and have failed to provide consistent clinical outcomes. Subsequently their use is not widespread and many clinics still rely on preparing sperm with much less rigour when compared with the overzealous care given to oocytes.

Of course the ultimate experiment is to isolate the handful of sperm that arrive in the Fallopian tube after coitus and examine their characteristics. Currently, these data do not exist but we can postulate that this handful of sperm would be highly motile, morphologically normal, contain a nucleus without DNA strand breaks and a membrane equipped with the appropriate receptors to traverse the male and female reproductive tracts and successfully interact with the oocyte vestments.

### Hyaluronic acid binding capacity

During sperm plasma membrane remodelling, specific receptors are created which facilitate sperm transport and fertilization, including the zona pellucida receptors and hyaluronic acid (HA) receptors which aid binding to the cumulus. It was shown in the late 1970s that a critical step in cumulus expansion is the deposition of a HA matrix and that the synthesis of this glycosaminoglycan was very important in the preparation of the mouse oocyte–cumulus cell complex for normal ovulation (Eppig, 1979). Hyaluronan has also been shown to be a major component of the human oocyte–cumulus complex (Dandekar *et al.*, 1992; Salustri *et al.*, 1999). Given the presence of this glycosaminoglycan in the reproductive tract (Yanagishita, 1994) and its positioning in the oocyte vestments it is logical that sperm expressing the HA receptor would be better equipped to achieve fertilization. The presence of a naturally occurring receptor translates to a diagnostic and selection tool that may discriminate which spermatozoa would at least bind to the oocyte vestments.

In the initial series of studies by Huszar and colleagues it was shown that sperm displaying HA binding capacity are more likely to have positive characteristics, namely that they are similar to those found bound to the zona pellucida, show low DNA fragmentation, decreased chromosomal aneuploidies, decreased levels of apoptotic marker proteins and normal morphology (Jakab *et al.*, 2005; Huszar *et al.*, 2006, 2007).

From the diagnostic viewpoint, HA binding ability of sperm has been recommended to be routinely tested before using ART. In a multicentre clinical trial, Worrilow *et al.* (2013) showed that pre-screened and selected patients with <65% HA binding efficiency before ICSI, exhibited slightly higher success rates with the use of this technology, but a significant reduction in miscarriage rates (3.3 versus 15.1% in controls). Unfortunately, other studies have shown inconsistent results. For example, Tarozzi *et al.*, (2009) showed that the proportion of sperm binding to HA was not correlated with fertilization, cleavage, percentage of good quality embryos, or miscarriage and pregnancy rates in couples undergoing IVF. Patients with clinical pregnancies had a percentage of HA-bound sperm similar to that found in patients with no pregnancy (Tarozzi *et al.*, 2009). In another study, HA-bound spermatozoa used for ICSI yielded a better embryo quality and cleavage rate although fertilization or pregnancy rates remained comparable with those treatments using spermatozoa conventionally selected (Parmegiani *et al.*, 2010). Another HA selection comparison with routine sperm preparation reported significantly higher fertilization rates after ICSI, while pregnancy rates were only moderately increased (Nasr-Esfahani *et al.*, 2008). The lack of studies has been highlighted by the recent Cochrane Database Review which concluded that evidence was insufficient to determine whether sperm selected by HA binding improve live birth or pregnancy outcomes in ART, and no clear data on adverse effects were available (McDowell *et al.*, 2014).

**Figure 2:**
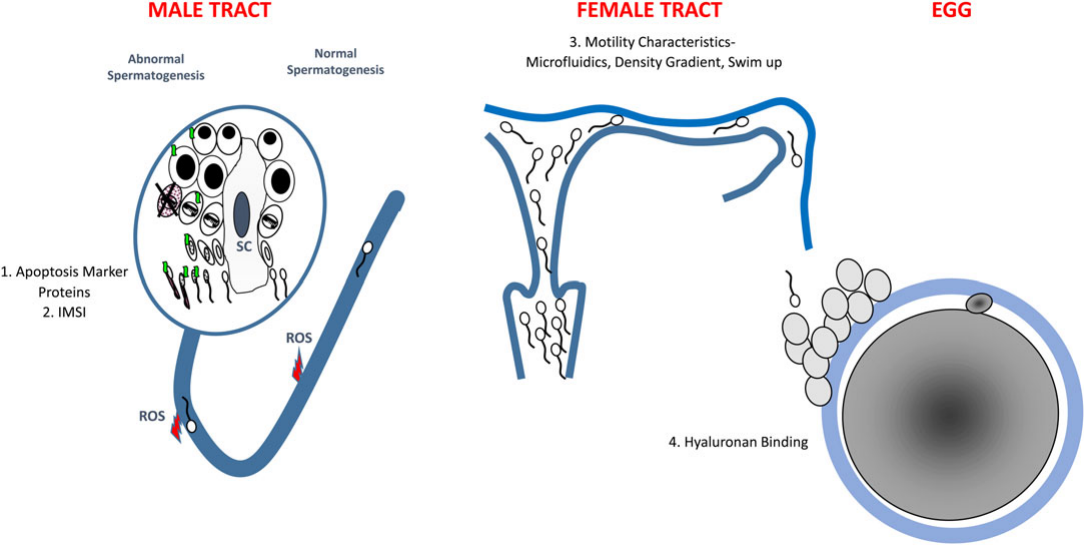
A schematic representation of how and where current sperm selection techniques focus on removing spermatozoa with individual issues in relation to nature. Four current sperm selection techniques are likened to those steps in spermatogenesis, sperm transport or egg interaction they impact. (1) Apoptosis marker proteins (e.g. Annexin with magnetic activated cell sorting) deselect spermatozoa that express apoptotic markers on the membrane after spermatogenesis. (2) Intracytoplasmic morphologically selected sperm injection (IMSI) removes sperm with abnormal morphology that arise after spermatogenesis. (3) Swim up, density gradients and new technologies such as microfluidics and electrophoresis separate spermatozoa based largely on their density or motility characteristics. (4) Spermatozoa that express a normal array of membrane receptors after spermatogenesis are selected based on their ability to bind to the cumulus cells or zona pellucida. One example is the Hyaluronan binding assay. ROS, reactive oxygen species.

Apart from the Worrilow *et al.* (2013) study, not many studies have evaluated the results obtained by the use of sperm selected by their ability to bind HA in clinical settings. Currently there are no HA threshold values established and widely accepted in order to predict the outcome of ART. This limits the value of estimating the proportion of HA-bound sperm in predicting IVF outcome (Nijs *et al.*, 2010, 2011). The clinical diagnostic value for HA binding theoretically attempts to mimic which sperm will bind to the cumulus cells (Fig. 2). As sperm binding to the cumulus–oocyte complex is one of the final steps in natural selection, the utility of this test would appear promising, however larger studies are urgently needed.

### Motile sperm organelle morphology examination

The rigorous journey for a sperm to arrive at the egg through the male and female reproductive tract would likely preclude sperm with abnormal morphology. It is evident however that sperm with poorer quality and abnormal morphology in other species can arrive at the site of fertilization (van der Horst *et al.*, 2011). In human a heterogeneous population of sperm is ejaculated with many sperm considered abnormal. In the early 1980s Roelf Menkveld established sperm morphology criteria that could be evaluated in order to improve the diagnosis and treatment of male infertility (Mortimer and Menkveld, 2001). Sperm morphology and hyperactivated motility also show a high correlation with the capacity of sperm to achieve tight binding to zona pellucida (Oehninger *et al.*, 1997). In the examination of the population of sperm it appears that morphology can be used as an indicator of infertility. However, what about the evaluation of a single spermatozoon? One of the main concerns in ICSI is the subjective selection of a spermatozoon based on the embryologist's gross evaluation under an optical magnification of ∼X400 (Souza *et al.*, 2010). This magnification is not sufficient to show subtle nuclear defects, with the risk, according to some authors, of having low success chances or even transmitting genetic and chromosomal diseases (Berkovitz *et al.*, 2006; Cassuto *et al.*, 2014). Would these subtle morphological anomalies preclude sperm from arriving at the egg *in vivo*? Mortimer *et al.* (1982) did show that some spermatozoa with abnormal head forms, but no other obvious signs of abnormality, could reach the uterus and oviducts.

In 2001, Bartoov *et al.* (2001) pioneered the development of a new sperm quality marker, the motile sperm organelle morphology examination (MSOME). This is based on a morphological analysis of isolated motile spermatozoa in real-time at a high magnification of up to X6600 providing information about both conventionally assessed morphological sperm alterations but also, more specifically, sperm head vacuole presence, size and location, as well as detailed characteristics of shape, acrosome, neck, tail, and other minor sperm structures.

Sperm vacuoles have frequently been associated with DNA fragmentation (Cassuto *et al.*, 2012) but this has also been questioned (Berkovitz *et al.*, 2006; Komiya *et al.*, 2013) given their variable number, size or distribution, and criteria to catalogue them.

The clinical impact of such defects on conventional semen parameters or other sperm quality markers, such as DNA fragmentation and aneuploidy, have been described in detail (Hammoud *et al.*, 2013). The advantage of using high-power microscopy for selecting spermatozoa is thought to be conveyed by avoiding selection of sperm with these defects (Garolla *et al.*, 2008; Avendano *et al.*, 2009).

As with many laboratory grown techniques, the use of MSOME as a diagnostic tool *per se* seems far from standardized, with different definitions available for a normal spermatozoon. The definition of normalcy initially described by Bartoov has been slightly modified by various author's opinions or data (Vanderzwalmen *et al.*, 2008; Cassuto *et al.*, 2009; Mauri *et al.*, 2010; Perdrix *et al.*, 2012). Moreover, information regarding the clinical impact of these sperm features is limited.

Probably, the lack of a predictive value of MSOME analysis is related to the fact that analysing the morphology pattern seen in an entire semen sample is not reflecting the likelihood of the individual sperm employed to fertilize the oocyte. It is also clear that the definition of sperm morphology and its assessment, even in an individual sperm, is a very controversial field (Eliasson, 2010).

Although some authors have recommended MSOME with ICSI to be systematically offered to all patients there are concerns that regular use is unwarranted, with unclear patient benefit for unselected males and even for specific indications (Bartoov *et al.*, 2003; Hazout *et al.*, 2006; Antinori *et al.*, 2008; Lo *et al.*, 2013). Moreover, another described benefit of MSOME and intracytoplasmic morphological sperm injection (IMSI) may be the reduction in the health related risks in the IMSI conceived children compared with regular ICSI (Cassuto *et al.*, 2014). The ability to detect subtle malformations in morphology may also be confounded by surrounding noise in the form of morphological polymorphisms and phenotypical differences without clinical relevance.

The studies comparing MSOME/IMSI procedures against routine ICSI show conflicting results in regard to numerous clinical outcomes, including fertilization, embryo characteristics, pregnancy, and live birth rates (Hazout *et al.*, 2006; Antinori *et al.*, 2008; Mauri *et al.*, 2010). Mauri *et al* (2010) suggested that IMSI may not be of benefit in improving the paternal component during early steps of fertilization (Mauri *et al.*, 2010). However, other studies have indicated that IMSI may impact on the later stages such as implantation, as higher pregnancy rates and diminished abortion rates were reported in couples that underwent IMSI when compared with couples that underwent routine ICSI (Bartoov *et al.*, 2003; Berkovitz *et al.*, 2006). The results presented in two meta-analyses, one comparing results from 357 IMSI cycles versus 349 routine ICSI cycles from three studies showed a significant improvement with IMSI in pregnancy and abortion rates but not in fertilization rates (Souza *et al.*, 2010). The most relevant information is available from a Cochrane review, concluding that the results from RCTs do not support the clinical use of IMSI while the evidence of effect on live birth or miscarriage and the evidence that IMSI improves clinical pregnancy is of very low quality (Teixeira *et al.*, 2013).

Perhaps this lack of evidence could arise because of questions relating to the ultimate resolution of the light microscope. The maximum useful magnification of an image is usually set at ∼1000 times. Magnifications higher than this will yield no further useful information or finer resolution of image detail, and will usually lead to image degradation. The numerous studies reporting IMSI must report whether adequate instrumentation was used as any aberrations seen at 5000× or 6600× may represent artefacts and not real structures.

### DNA/chromatin integrity

DNA integrity in sperm has been by far the most studied molecular feature in sperm. Although there is no argument that sperm with abnormal DNA/chromatin integrity exist in ejaculates the clinical utility of this information is controversial. Intrauterine insemination (IUI) results come closest to mimicking the sperm journey when compared with natural conception (Fig. 1). Interestingly, the most intriguing information regarding sperm DNA/chromatin integrity is that they correlate best with outcomes of natural conception and IUI (Bungum *et al.*, 2007) and seemingly less with IVF and ICSI. In addition, data exist showing that high levels of DNA fragmentation are associated with recurrent pregnancy loss (Carrell *et al.*, 2003; Ribas-Maynou *et al.*, 2012; Ruixue *et al.*, 2013). It therefore seems possible that either DNA compromised cells have issues arriving at the egg and fertilizing or, when they do fertilize, the fetus may be compromised in development.

The origin of sperm DNA fragmentation can be multiple, endogenous or exogenous, and includes defective spermatogenesis, abortive apoptosis, protamine defects, reactive oxygen species attack, different medical pathologies (cancer, varicocele, infections), age, life recreational habits or working conditions, etc., with sperm exhibiting few DNA repair mechanisms, against the demonstrated ability of the oocytes to repair limited damage (Sakkas *et al.*, 2002; Seli and Sakkas, 2005; Sakkas and Alvarez, 2010; Esbert *et al.*, 2011; Meseguer *et al.*, 2011). Several tests are available in order to measure sperm DNA damage, and their description has been discussed extensively elsewhere (Schlegel and Paduch, 2005; ASRM guideline, 2013; Ribas-Maynou *et al.*, 2013). This measurable damage may include single or double strand DNA damage or minor chromatin anomalies. The majority of current tests are only showing gross anomalies and are not capable of picking up minor anomalies. In addition, the lack of standardization among laboratories negatively contributes to the implementation of this technique worldwide. These confounders may be influencing the results provided by some reports, regarding the effect of DNA damage in sperm function, and has resulted in the absence of an accurate and useful standard analysis to predict reproductive success by measuring DNA damage.

Apart from these facts, it seems there is a general agreement regarding, first, the link between sperm DNA fragmentation and male infertility, and second, the possibility of inadvertently microinjecting a DNA-damaged sperm during ICSI, resulting in worse embryo quality and lower reproductive results, either in pregnancy achievement or on miscarriage rates (Evenson *et al.*, 1999; Fernandez *et al.*, 2000; Collins *et al.*, 2008; Bungum, 2012), as well as the future child's health (Ahmadi and Ng, 1999; Zini and Libman, 2006; Fernandez-Gonzalez *et al.*, 2008; Zini *et al.*, 2008). To what extent DNA fragmentation decreases reproductive results is undetermined and conflicting results are available (Payne *et al.*, 2005; Niederberger, 2011) although it seems sufficiently justified to select DNA-intact sperm for use in ART to improve results.

### Apoptosis

In the adult testes, programmed germ cell death or apoptosis plays a pivotal role in sperm output. Withdrawal of gonadotrophins and testosterone further enhances the degeneration of germ cells in the testis, indicating that it is a major mechanism to select against germinal cells that show early defects (Sinha Hikim and Swerdloff, 1999). Apoptosis in the testes is, however, complicated by the fact that once a sperm reaches the spermatid stage, true apoptosis is difficult to trigger. Infertile males display a varying percentage of ejaculated sperm cells positive for apoptotic marker proteins, including Fas, the Bcl family, caspases, cleaved poly ADP ribose polymerase (Parp), p53, etc. (Sakkas *et al.*, 1999, 2002, 2003; Oehninger *et al.*, 2003; Cayli *et al.*, 2004; El-Domyati *et al.*, 2009; Mahfouz *et al.*, 2009; Enciso *et al.*, 2012).

Although frequently related to other abnormalities detectable by light microscopy, it seems that sperm cells with apoptotic features can remain normally shaped. The potential interest of removing apoptotic sperm relies on the fact that they may still be able to fertilize an oocyte. In particular, during an ICSI treatment, the sperm may be forced inside the oocyte after being selected by the operator and this could adversely affect embryo or fetal development.

Where these apoptotic cells originate or why they are not efficiently removed during spermatogenesis or transport through the reproductive tract naturally remains unclear (Sakkas *et al.*, 2003). Recent evidence suggests that the free radical-mediated phenomena may be more crucial in relation to apoptosis, as prolonged exposure of human spermatozoa to phenylalanine resulted in the stimulation of apoptosis via mitochondrial superoxide generation and the activation of intracellular caspases (Houston *et al.*, 2015). This sheds new light on the relationship between sperm and apoptosis as some sperm may not have the intrinsic mechanisms to undergo apoptosis.

One apoptotic marker that has come under more scrutiny in relation to sperm selection is Annexin V. Numerous reports have shown that ejaculated spermatozoa experience changes consistent with apoptosis-like features. These have been observed mainly in infertile males and include phosphatidylserine externalization, mitochondrial membrane potential disruption, activation of caspases-3 and/or DNA fragmentation, which are all phosphatidylserine-related events and early indicators of apoptosis (Grunewald *et al.*, 2009; Lee *et al.*, 2010; Tavalaee *et al.*, 2012).

Phospholipid phosphatidylserine (PS) is normally located in the inner leaflet of the sperm plasma membrane. This phospholipid has high affinity for Annexin V that is a phospholipid-binding protein of 35–36 kDa lacking the ability to pass through an intact sperm membrane. Consequently, any binding between annexin V (Glander and Schaller, 1999) and PS needs to have PS on the outer surface of the sperm plasma membrane, indicating that the membrane integrity is compromised.

Magnetic activated cell sorting (MACS) of apoptotic sperm is based on the availability of colloidal superparamagnetic microbeads (50 nm diameter) conjugated with annexin V able to bind to PS (reviewed by Said *et al.*, 2008). This technique has been utilized to separate dead and apoptotic spermatozoa (Grunewald *et al.*, 2001; Said *et al.*, 2008; Lee *et al.*, 2010). Sperm cells are labelled with annexin-V magnetic beads and passed through a column, which is placed in the magnetic field of a MACS separator.

Several research groups are currently studying the possible clinical application of this method to improve the fertilization potential of sperm and the outcome of ART. They have shown that spermatozoa of infertile patients contain higher levels of activated apoptosis signalling than donors and therefore lower chromatin decondensation rates (Grunewald *et al.*, 2009). Other groups have investigated the efficiency of MACS on its own or in combination with DGC (density gradient centrifugation). Lee *et al.* (2010) found that the levels of spermatozoa with apoptotic markers in idiopathic infertility (externalization of phosphatidylserine and DNA fragmentation) decreased after DGC, but the combination DGC + MACS presented even better results.

To date, some reports have described the use of these techniques and the achievement of the first newborns, although the data presented are limited, and there are few studies adequately addressing the benefits of MACS to improve clinical take home baby rate by selecting non-apoptotic sperm cells compared with standard sperm selection methods (Dirican *et al.*, 2008; Polak de Fried and Denaday, 2010; Rawe *et al.*, 2010). A recent meta-analysis by Gil *et al.* (2013) showed some improvement in pregnancy rate, however there were no differences reported in implantation and miscarriage rates. Romany *et al.* (2011) has evaluated the relevance of MACS in the results of IUI due to apoptotic spermatozoa and shown that they are more frequent in infertile men. The results show that IUI with selected non-apoptotic sperm by MACS shows a trend to improve ongoing pregnancy rates with raw samples despite decreasing total progressive motility.

### Other sperm selection strategies under investigation

A number of other methodologies are being adapted as sperm markers. These range from simple to more complex procedures. For example, Raman Spectroscopy has been identified as a means of identifying specific cell traits, including sperm DNA damage (Huser *et al.*, 2009; Meister *et al.*, 2010; Mallidis *et al.*, 2011, 2014; Sanchez *et al.*, 2012). Spectra from different regions of the sperm have been described, including DNA within the sperm head, based mainly on changes in a peak at 1092 cm-1 (suggested to be the DNA backbone). The advantage of this technology is that the nuclear DNA status can be checked on live sperm. A number of studies have also verified the Raman information with different DNA fragmentation evaluation methods (Mallidis *et al.*, 2011; Sanchez *et al.*, 2012). Although clinical information is lacking using this technique, it has been used to identify traits in the spermatozoa bound to the zona pellucida. This technique may therefore be useful in the future to detect normal functional sperm for ICSI (Liu *et al.*, 2013).

Sperm membrane integrity or viability can also be checked by the use of hypo-osmotic solutions, due to the semi-permeable features they exhibit. The hypo-osmotic swelling test (HOST) has been employed historically in cases of sperm samples with 100% immotile cells, such as those from patients with Kartagener's syndrome (Jeyendran *et al.*, 1984, 1992; Ramu and Jeyendran, 2013).

Recently, it was also reported that the HOST test can identify individual spermatozoa with minimal DNA fragmentation (Stanger *et al.*, 2010), and with traits of apoptosis, abnormal head morphology, nuclear immaturity, or membrane damage (Bassiri *et al.*, 2012). Initial clinical results support the need to validate this technique prospectively, since the use of HOST selected sperm to microinject has been reported to be beneficial for implantation and pregnancy rates in patients with only immotile cells (El-Nour *et al.*, 2001), and its role in recurrent miscarriage has also been demonstrated by several clinical studies (Buckett *et al.*, 1998; Patankar *et al.*, 2001; Bhattacharya, 2010). For testicular sperm, fertilization rates have been shown to be better, yielding increased pregnancy rates in prospective and randomized trials (Sallam *et al.*, 2001).

Of the numerous other methods investigated sperm membrane charge has also been used. Two methodologies are the Zeta potential (Chan *et al.*, 2006) and an electrophoretic chamber (Aitken *et al.*, 2011). Although promising (Ainsworth *et al.*, 2007), neither has been tested to see if they convey significant clinical improvement.

Unfortunately, of all the techniques developed to date there is no clear sperm selection technique that has provided conclusive evidence of improving pregnancy rates in large clinical trials.

## Massive molecular analysis techniques: the ‘Omics’ and the evaluation of sperm quality

The previous techniques have used simpler targeting methods to identify a sperm diagnostic/selection marker. More powerful global screening techniques can be adopted to examine sperm populations that are separated according to their ability to achieve a pregnancy. The ‘Omics’ technologies are disciplines that include the study of the events and interactions of cellular structures and processes from DNA to biological function, i.e. from DNA and genes to metabolites in a global manner.

### Transcriptomics of sperm and male fertility

Although the functional significance of messenger RNA (mRNA) in mature spermatozoa has been extensively debated (Miller *et al.*, 2010), it is becoming clear that specific paternal mRNAs are necessary from the first embryo cleavages until the embryo activates its own genome (Krawetz, 2005).

It has been hypothesized that the molecular requirements for semen samples able to achieve a pregnancy differ for each type of assisted reproduction procedure, and there are fewer molecular requirements when the reproductive techniques are more invasive, such as ICSI, meaning that the machinery needed for sperm function is minimal (Garrido *et al.*, 2013).

Using this concept the different gene expression profiles were examined for IUI, IVF and ICSI depending on whether pregnancy was achieved or not (Garrido *et al.*, 2013). A number of specific genes, biological processes, cellular components, etc. have been demonstrated to be differently expressed depending on accomplishing pregnancy, including genes newly described as related to male fertility depending on the method evaluated, either IUI, IVF or ICSI. Interestingly, some of these genes were found to be important for reproductive results in all three ARTs, thus being of special interest in order to unveil a role in sperm function (Garrido *et al.*, 2013).

Particular features of the spermatozoon have also been related to mRNA expression profiles. In this sense, differences between asthenozoospermic patients and normal controls were seen, identifying up to 19 genes (Jodar *et al.*, 2012), and linking sperm mRNA expression with clinical results. Bonache *et al.* (2012) demonstrated that sperm mRNA may be a parameter able to predict the results of IUI with donor sperm (Bonache *et al.*, 2012) after comparing gene expression patterns of 68 normozoospermic donors and describing significant differences in the expression of individual genes between donors with the highest and lowest pregnancy rates.

Several molecules were described to be differentially expressed between the high and low pregnancy rate groups, and also several molecules were described to be present or absent in one group, being considered, respectively, as potential fertility or infertility markers. The differences described between expression profiles from sperm samples achieving pregnancy and failing, were suggested as a possible future diagnostic test in order to complement basic sperm analysis, although prospective studies of the diagnostic and clinical usefulness are still necessary. Using a similar paradigm a test for endometrial receptivity, analogous to the one proposed for sperm, has been developed to potentially improve success rates in implantation failure cases (Ruiz-Alonso *et al.*, 2013; Blesa *et al.*, 2014).

### Proteomics of sperm and fertility

A similar approach can be applied to sperm cells at the proteomic level (Aitken *et al.*, 2007; Aitken and Baker, 2008). A large number of proteins have been identified to date and may play a role in male fertility (Oliva *et al.*, 2009; du Plessis *et al.*, 2011). Proteomic analysis techniques provide information regarding functions developed by sperm proteins, also identifying post-translational modifications able to affect protein and subsequently sperm function. Moreover, proteomics permits the targeting of the whole cell and subcellular compartments (Aitken and Baker, 2008; Amaral *et al.*, 2014; Castillo *et al.*, 2014).

Studies describing sperm proteomes or protein maps have described the whole sperm extract from fertile, subfertile, normozoospermic and donor males (Johnston *et al.*, 2005; Martinez-Heredia *et al.*, 2006; Li *et al.*, 2007; de Mateo *et al.*, 2013), and subcellular fractions including heads, tails, fibrous sheaths or membranes (de Mateo *et al.*, 2011; Amaral *et al.*, 2014). Studies have also used so-called comparative proteomic analysis, where the results yielded a potential list of proteins differentially found after comparing sperm samples failing or achieving fertilization, and with specific sperm traits such as motility or morphology, or even capacitation (Pixton *et al.*, 2004; Frapsauce *et al.*, 2009; Xu *et al.*, 2012). However, as yet, despite the immense promise of proteomics to provide clear diagnostic/prognostic information clear robust markers are yet to be identified.

### Metabolomics of sperm and fertility

Metabolome analysis exhibits some advantages compared with the above ‘omics’ approaches. Theoretically it targets the end products of expression, translation, and protein modification, including protein function, therefore providing higher sensitivity to be employed as sperm function biomarkers.

Nevertheless, the detection and measurement of these compounds may require extremely expensive and sophisticated techniques such as high pressure liquid chromatography, mass spectroscopy and nuclear magnetic resonance. Abnormal spermatogenesis has been investigated using this approach (Aaronson *et al.*, 2010) as well as seminal plasma from fertile and infertile men (Gupta *et al.*, 2011).

Among metabolites involved in sperm function, sperm membrane lipids are especially interesting due to the properties they confer on the sperm plasma membrane, and their involvement in events that lead to successful fertilization, capacitation, interaction with the oocytes (Flesch and Gadella, 2000; Kawano *et al.*, 2011) and freezability (survival to a freezing/thawing processes) (Maldjian *et al.*, 2005; Kaeoket *et al.*, 2010; Beirao *et al.*, 2012). To date the lipid profile in subfertile males has also been analysed (Maldjian *et al.*, 2005; Garcia *et al.*, 2011; Beirao *et al.*, 2012). New lipid compounds have been found in these samples, together with relevant molecules previously linked with sperm function. However, its usefulness to improve the diagnosis of male infertility and forecast the outcomes of ART needs to be addressed in further studies.

### Predicting success

**Figure 3:**
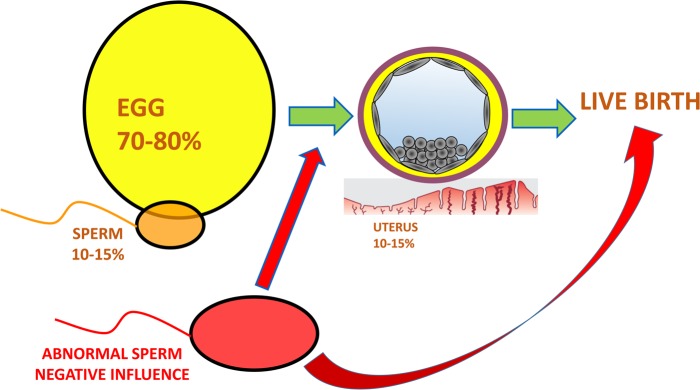
A schematic representing the hypothetical relative contributions of the egg, sperm and uterus to a successful live birth. The relative contribution of the sperm to a successful live birth can be hypothesized to be 10–15%. Aneuploidy and maternal age studies show that overall the egg's contribution is greater and could be hypothesized to be 70–80%, however when the paternal component is abnormal (red) it can drastically affect the ability of an embryo to reach the blastocyst stage and/or a fetus to develop and result in a live birth.

In a sense, our greatest challenge to creating predictive tests or models is the multifactorial nature of fertility. We are not only dealing with the many attributes of the spermatozoon but also a significant impact from the egg and uterus (Fig. 3). A multiparametric test to assess spermatozoa is likely to utilize several biomarkers of sperm function. In this sense, our current approach to analyse the link between biomarkers and clinical results must undergo a quantum leap in thinking and utilize a more holistic approach. This should encompass, but not be limited to, several of the following criteria. The first of these is a robust assessment using appropriate methods. Currently there is considerable focus on the use of high quality and robust methods in all aspects of research (Masca *et al.*, 2015). Reproductive medicine is no exception. Poor methodology has plagued andrology (Barratt *et al.*, 2011) and high quality methods which are repeatable and reliable are an absolute necessity for the development of clinically useful diagnostic tools. Second, is a vigorous assessment of putative tools including their place in the diagnostic and treatment pathway. All too often sperm biomarkers are not rigorously assessed. There is an absolute requirement to examine their effectiveness in different clinical scenarios in a variety of clinical settings. Additionally, how and where these biomarkers fit in the diagnostic/treatment pathway needs to be ascertained. The evaluation of diagnostic tests is subject to renewed critical examination and they can be used as replacement, triage or add-on with their usefulness being dependent on a large number of factors (Lefievre *et al.*, 2007; Horvath *et al.*, 2014). Truly effective biomarkers will stand up to this comprehensive examination and thus the discipline will benefit with clinically validated and useful tests. A third criterion is time. In other disciplines the development of biomarkers takes considerable time and resources (Duffy *et al.*, 2015). In reproductive medicine we often translate initial findings into clinical practice with little critical appraisal, which undoubtedly sets the field back (Harper *et al.*, 2012).

## Conclusion

### Identifying the right sperm for ART and assessing their physiological function

The current, modern tests of sperm function or selection have consistently failed to provide clear evidence of any real value in improving success rates of ART. This has led to frustration and scepticism in their clinical utilization. Historically, *in vivo* attributes of sperm were examined to identify characteristics of the successful cells (Gould *et al.*, 1984) but more modern research has failed to follow this approach. This paper has presented the thesis that identifying the attributes of the spermatozoa that are able to successfully navigate the female reproductive tract and arrive at the site of fertilization will allow us to also identify the best cells for selection in ART. These cells are after all the ones that are able to surpass all the natural challenges put in place by millions of years of evolution. Understanding their characteristics (DNA/chromatin, membrane integrity, morphological traits and ‘omics’ profiles) will allow us to develop functionally relevant diagnostic and sperm selection strategies to maximize reproductive success. We are now able to action this approach, as technologies applied to spermatozoa are increasingly sophisticated and can examine very low numbers, even single cells, for example by electrophysiology (Frimat *et al.*, 2014; Mansell *et al.*, 2014; de Wagenaar *et al.*, 2015), allowing detailed assessment of the selected populations to a level not previously possible.

Even so, identification and assessment of the physiological attributes are likely to need to be tailored to the type of ART used. For example the diagnostic profile of a cell used for IUI is unlikely to be equal to that required for ICSI (Fig. 2). Once developed any sperm selection strategy needs to be applied in the context of ‘personalized medicine’ whereby they are linked with a diagnostic component. Many of the current studies try to adapt the sperm selection strategy to all infertility patients. This is inappropriate. It is interesting that when HA binding was restricted to a specific subset of patients with low binding, the same patients showed the greatest benefit (Worrilow *et al.*, 2013).

It is often overlooked that, in addition to the oocyte and uterus, sperm contribute a specific component to establishing a successful pregnancy. More importantly, the poorer the quality of the fertilizing cell the greater the negative paternal contribution and lower the chance of achieving a live birth (Fig. 3). Understanding and being able to identify sperm that exhibit the optimal physiological features is likely to be a key component in improving the chance of a live birth, in particular for couples undergoing ART. Looking forward we need to heed the lessons of the past and match research on ART with complementary basic questions addressing the intricacies of sperm transport and function *in vivo*.

## Authors' roles

All authors were involved in the conception of the review, and writing the initial and final drafts of the manuscript.

## Funding

Work in C.L.R.B.'s laboratory is funded by TENOVUS (Scotland), University of Dundee, Medical Research Council (MRC grant numbers 4190 and 12492) and NHS Scotland. Funding to pay the Open Access publication charges for this article was provided by the Medical Research Council.

## Conflict of interest

C.L.R.B. is Editor in Chief of Molecular Human Reproduction and Chairperson of the WHO Expert Working Group on male infertility (2012–2016). D.S. serves on the Scientific Advisory Board of ORIGIO. M.R. and N.G. have no conflicts of interest.
